# Group emotion awareness in socially shared regulation of learning: a conceptual framework and process model

**DOI:** 10.3389/fpsyg.2026.1878525

**Published:** 2026-10-14

**Authors:** Jiawen Chen, Xiangdong Chen, Sihan Song, Chen Guo, Chunlei Liang, Ruijun Zhao

**Affiliations:** 1 School of Artificial Intelligence, Shanghai Normal University Tianhua College, Shanghai, China; 2 Faculty of Education, East China Normal University, Shanghai, China; 3 College of Information and Mechanical & Electrical Engineering, Shanghai Normal University, Shanghai, China; 4 WisFutura, Shanghai Zhiyun Weilai Technology Co., Ltd., Shanghai, China; 5 College of Future Teachers, Hefei Normal University, Hefei, Anhui, China; 6 Hefei Huafujunyuan Primary School, Hefei, Anhui, China; 7 Pinghu Normal College (School of Education), Jiaxing University, Jiaxing, Zhejiang, China

**Keywords:** collaborative learning, CSCL, emotion awareness, emotion regulation, group emotion awareness, learning analytics, scaffolding, socially shared regulation

## Abstract

In collaborative learning environments, fostering positive emotional experiences and effective emotional management is of paramount importance for creating a supportive and productive learning atmosphere. A fundamental prerequisite for achieving this goal is the cultivation of timely and effective emotional awareness within collaborative groups. A growing body of research suggests that group emotional awareness during the collaborative process is critical to both the quality of team interaction and Socially Shared Regulation of Learning (SSRL). Drawing upon the characteristics of SSRL, this study proposes a conceptual framework for group emotional awareness by analyzing the specific emotional information required across different stages of shared regulation and their respective functional dimensions. To integrate emotional awareness into the SSRL process, this paper further constructs a corresponding process model and achieves its practical embedding through the design of instructional scaffolding and the integration of existing technological tools. Addressing the research question of how to implement group emotional awareness models in collaborative scenarios, this study adopts a theory-driven evidence-chain synthesis approach to integrate representative empirical findings. It synthesizes key conditions and process evidence—ranging from the externalization of emotional cues and shared interpretation to the formation of shared problem representations—thereby elucidating the feasible paths and applicable boundaries of the proposed model in supporting learners to enhance emotional awareness and conduct emotional attribution inquiries. Moving beyond previous research that predominantly focused on emotional measurement techniques or tool development, this paper advances the implementation framework for group emotional awareness in collaborative learning from the dual perspectives of theoretical modeling and evidence-chain support.

## 1 Introduction

Emotion plays a pivotal role in collaborative learning, not only facilitating team interaction and cooperation but also exerting a profound impact on individual learning outcomes. Emotional awareness among collaborators is a vital factor in maintaining interpersonal interaction and fostering a positive socio-emotional atmosphere within the group (Ucan and Webb, 2015). Given that collaborative processes involve a wealth of implicit emotional information, learners often struggle to perceive shifts in emotional states in a timely and effective manner on their own (Kreijns et al., 2003). Consequently, how to dynamically promote emotional awareness during collaboration to enable individuals or groups to engage in effective subsequent regulated learning has become a critical issue in computer-supported collaborative learning (CSCL), offering potential solutions to various challenges inherent in collaborative learning (Metallinou and Narayanan, 2013).

In recent years, research on emotional awareness within CSCL has increasingly pivoted toward a group-centric perspective. This approach focuses on how members of a collaborative group understand one another’s emotions and reactions (Xolocotzin Eligio et al., 2012), thereby enhancing learners’ understanding of the team’s collaborative dynamics (Elfenbein, 2007). Group Emotion Awareness (GEA), on one hand, signifies the elevation of emotional perception from the individual to the group level (Chen et al., 2024). On the other hand, it originates from the research perspective of group affect or group affective tone (Collins et al., 2013): group-level states can often be characterized across multiple dimensions—cognitive, affective, behavioral, and social interaction. Within the group awareness framework of CSCL, the “affective” dimension is highlighted to form a continuous perception and shared understanding of group emotional responses and their fluctuations—namely, GEA. Concurrently, it draws upon the perspective of Group Awareness (Bodemer and Dehler, 2011), focusing on the internal emotional states (affective reactions) and psychological activities of group members (Bodemer et al., 2018).

Group Emotion Awareness exerts multifaceted positive effects on collaborative learning. First, it helps improve overall team engagement, as positive group emotional states can significantly enhance both the affective and cognitive investment of members, prompting them to adopt adaptive regulation strategies to overcome academic challenges and complete tasks efficiently (Vega, 2014). Second, emotional awareness can mitigate misunderstandings and conflicts among members, creating a positive socio-emotional climate. This not only strengthens team cohesion and collective motivation (Rogat and Linnenbrink-Garcia, 2011) but also facilitates individual self-monitoring and regulation through socio-emotional control and mutual encouragement, thereby sustaining team momentum (Winnie and Hadwin, 1998). Finally, GEA effectively reduces cognitive load associated with negative emotions (van der Meer et al., 2010) and enables emotional sharing beyond shared thinking, which inspires learners to explore diverse perspectives and further promotes the development of shared cognition and metacognition when tackling high-difficulty learning tasks (Hurme et al., 2009).

Currently, much of the research on emotional awareness focuses on designing specific Emotional Awareness Tools (Wang et al., 2019; Lavoué et al., 2020) or on technical and strategic studies for emotion mining and affective computing (Salian et al., 2021; Yuan, 2022). Few studies have addressed theoretical frameworks for emotional awareness, particularly at the group level. This theoretical vacuum leaves the transition from emotional awareness to appropriate learning regulation and changes in strategy selection obscured. Theoretically, many studies on GEA in collaborative learning are rooted in the perspective of Socially Shared Regulation (Järvenoja et al., 2013). The evolution of Socially Shared Regulation of Learning (SSRL) theory allows researchers to explore the impact of emotional awareness on collaborative learning from diverse angles (Järvelä et al., 2015).

Although existing research has proposed various emotional models (Feidakis et al., 2013), emotion regulation models (Gross, 2015), and group awareness models (Li et al., 2019), these are often oriented toward individual emotional mechanisms, general emotion regulation processes, or the cognitive/behavioral awareness of group states. There remains a lack of a model specifically designed to promote GEA throughout the collaborative learning process. Under the guidance of such a model, researchers could enhance emotion mining and attributional explanation during collaboration, while providing visualized awareness to facilitate subsequent collaborative regulation behaviors. This gap directly leads to a lack of stable theoretical guidance for the development of GEA tools, making it difficult to fully reflect the dynamic changes in individual and group emotional states or to support the regulatory function of emotion as social information in team interactions (Van Kleef, 2009) and the effective integration of multimodal emotion recognition technology in educational settings (Akçay and Oğuz, 2020).

Therefore, from the theoretical perspective of Socially Shared Regulation of Learning (SSRL), this study aims to systematically construct a theoretical framework and application model for Group Emotion Awareness. This effort seeks to address the insufficient theoretical support for group-level emotional attribution and the lack of deep integration with the collaborative process in current research. The goal is to explore how effective technological scaffolding can achieve group-level emotional awareness and transform it into a driving force for team regulated learning.

The primary content of this research includes: first, integrating Achievement Emotion Theory with SSRL theory to construct a multidimensional conceptual framework for GEA, clarifying its constituent dimensions and elements; second, designing and developing a process model and supporting instructional scaffolding that embed emotional awareness throughout the entire collaborative lifecycle (start, process, and end); finally, through a review of representative empirical evidence chains, this paper systematically organizes key conditions and process evidence—including the externalization of emotional cues, shared interpretation, the formation of shared problem representations, and the transitions between self-regulated learning (SRL), co-regulated learning (CoRL), and SSRL regulatory modes—thereby providing a theoretically grounded and practical framework for implementing the GEA model in authentic collaborative learning scenarios.

## 2 Literature review

### 2.1 Current state of research on emotion awareness

Emotion awareness refers to learners’ capacity to recognize, understand, and interpret their own emotions and those of others. It involves not only identifying emotional states, but also understanding how these emotions are connected to thoughts, behaviors, learning situations, and subsequent regulation. From a developmental and learning sciences perspective, cognition, social interaction, and emotion are deeply intertwined; emotions play an important role in learning, cognitive development, motivation, and social participation (Pekrun, 2011; Lantolf and Swain, 2019).

Existing studies generally define emotion awareness as an attentional and interpretive process through which individuals become conscious of their own and others’ emotions (Rieffe et al., 2008; Feidakis et al., 2014b). Brackett and Rivers further emphasize that emotion awareness requires individuals to attend to emotions and understand the relationships among emotions, thoughts, and behaviors (Brackett and Rivers, 2014). Accordingly, emotion awareness in learning contexts should not be limited to the perception of emotional states. It should also include the interpretation of why these emotions occur and how they may inform subsequent regulation.

#### 2.1.1 Perception of emotions

The perception of emotional states provides the basic informational foundation for emotion awareness (Feidakis et al., 2014b). Prior studies on emotional theories and emotion awareness tools have commonly adopted three types of emotion models: basic emotion models, dimensional emotion models, and eclectic or context-specific emotion models (Scherer, 2005; Ez-zaouia et al., 2020).

Basic emotion models describe emotions as discrete and recognizable categories, such as anger, fear, joy, sadness, disgust, and surprise, and have been widely used in facial-expression-based emotion recognition (Ekman and Friesen, 1978). Dimensional models, such as Russell’s circumplex model of affect, represent emotions along continuous dimensions such as valence and arousal, making them useful for quantifying and visualizing emotional states (Russell, 1980). In contrast, eclectic or context-specific models select emotion labels according to specific research goals. For example, Kort et al. (2001) learning-cycle model connects emotions such as anxiety, confidence, frustration, enthusiasm, and curiosity with different phases of learning.

These models provide useful ways to detect, classify, and visualize learners’ emotional states (Ez-zaouia et al., 2020; Lavoué et al., 2020; Zeng et al., 2021). However, for collaborative learning research, merely identifying whether learners feel anxious, frustrated, confused, or engaged is not sufficient. Emotional states become educationally meaningful only when they are interpreted in relation to learning tasks, social interaction, perceived control, task value, and collaborative challenges (Bakhtiar et al., 2018; Järvenoja et al., 2020; Kazemitabar et al., 2022). Therefore, emotion awareness in computer-supported collaborative learning (CSCL) needs to move beyond emotional visibility toward emotional interpretation.

#### 2.1.2 Perception of the causes of emotions

In CSCL contexts, emotion awareness is a prerequisite for emotion regulation, but its function extends beyond the emotional level itself (Boden and Thompson, 2015). When learners become aware of their emotions during learning, they often ask why these emotions arise. Such attributional inquiry may lead them to identify cognitive difficulties, motivational problems, social conflicts, task-value concerns, or environmental constraints (Järvenoja and Järvelä, 2009). Based on these interpretations, learners may further engage in regulated learning across cognitive, metacognitive, motivational, emotional, and behavioral dimensions (Boekaerts, 2011; Hadwin et al., 2017).

Achievement Emotion Theory, especially Pekrun’s Control-Value Theory of Achievement Emotions, provides a useful theoretical basis for explaining the causes of emotions in academic settings. Achievement emotions refer to emotions directly related to achievement activities or achievement outcomes (Pekrun, 2006). They include activity-related emotions, such as enjoyment, boredom, frustration, or anger during learning, as well as outcome-related emotions, such as hope, anxiety, pride, or shame regarding achievement results (Pekrun et al., 2002, 2006). According to Control-Value Theory, these emotions are shaped by learners’ appraisals of control and value in specific learning situations (Pekrun, 1992; Pekrun and Perry, 2014).

This perspective is particularly relevant to collaborative learning. In CSCL environments, learners frequently experience emotions related to task difficulty, peer interaction, goal alignment, responsibility distribution, and group progress (Bakhtiar et al., 2018). These emotions are not merely individual psychological reactions; they may signal problems in the collaborative process and provide cues for subsequent regulation (Järvelä et al., 2016b; Hadwin et al., 2018). Therefore, the present study adopts Control-Value Theory as the theoretical foundation for constructing a Group Emotion Awareness model (Pekrun, 2006). This model aims not only to support the perception of emotional states within collaborative groups, but also to help learners and groups interpret the causes of emotional changes and transform emotional cues into objects of shared regulation.

### 2.2 Theoretical perspective of socially shared regulation of learning (SSRL)

Socially Shared Regulation of Learning (SSRL), a concept pioneered by Hadwin et al. (2011), refers to the process in which collective members—through shared participation in phases such as task understanding, goal setting and planning, monitoring the collaborative process, and evaluation and reflection—regulate collective adaptation across cognitive, metacognitive, motivational, and emotional dimensions to achieve or maintain a shared understanding. Specifically, the cognitive dimension encompasses the knowledge and skills required for scientific problem-solving, inquiry, and critical thinking. The metacognitive dimension involves the knowledge and regulation necessary to understand and control one’s own cognitive processes. The motivational dimension entails regulating the drive required to sustain scientific thinking and deep reasoning, including the beliefs and attitudes that influence the use and development of an individual’s cognition and metacognition (Schraw et al., 2006).

Within collaborative learning, regulation may occur across different social levels, including self-regulated learning (SRL), co-regulated learning (CoRL), and socially shared regulation of learning (SSRL). SRL primarily involves individuals regulating their own learning, whereas CoRL involves temporary or transitional support in which one or more members guide or support another member’s regulation. SSRL, in contrast, involves the collective regulation of shared goals, processes, and outcomes by the group as a whole. These regulatory modes may coexist and dynamically shift in response to changing task and interactional demands (Järvelä and Hadwin, 2013; Järvelä et al., 2016a; Hadwin et al., 2017).

In the field of learning sciences, research on regulatory needs within the emotional dimension is rapidly expanding (Sinatra et al., 2014). During collaboration, learners may encounter various emotional and motivational challenges that can negatively impact team interaction and engagement (Järvenoja and Järvelä, 2009). When these impacts occur, emotion regulation takes place—either consciously or unconsciously—as an individual attempts to alter their own or their peers’ emotional states. While the exact role of emotion awareness in activating the goals of emotion regulation remains to be fully elucidated, research by Barrett et al. (2001) and Samson et al. (2012) underscores that emotion awareness is undoubtedly a critical factor in successful emotion regulation.

Within the SSRL theoretical framework, regulatory strategies in the emotional dimension rely on learners’ awareness of both individual and group emotions during the collaborative process. Emotion awareness is the primary prerequisite for emotion regulation, which, in turn, depends on socially shared regulation within a socio-emotional context. It is further influenced by other factors such as cognition, metacognition, motivation, behavior, and social interaction. Consequently, the study of emotion awareness necessitates the robust theoretical support provided by Socially Shared Regulation of Learning.

Despite the wealth of existing research on affective computing technologies and individual-level emotion awareness, two core challenges remain at the group level: first, the lack of an integrative framework oriented toward collaborative contexts that can systematically characterize the causes of group emotions and the logic of attribution; second, the absence of an operational path to deeply integrate emotion awareness information with the dynamic phases of SSRL (task understanding, planning, monitoring, and reflection). These challenges directly hinder the theoretical guidance and instructional implementation of Group Emotion Awareness (GEA) tools. Accordingly, this study proposes three research questions focusing on the composition of GEA (RQ1), the mechanisms and models for embedding it into the collaborative process (RQ2), and the key implementation points and applicable boundaries in authentic collaborative scenarios (RQ3).

Accordingly, the aim of this paper is to develop a theoretically grounded framework and process model for Group Emotion Awareness in collaborative learning, and to clarify how the proposed model can be operationalized and supported by existing empirical evidence within the SSRL process. To address this aim, three research questions are proposed:

*RQ1*: What constitutes Group Emotion Awareness?

This question primarily concerns the dimensions (or aspects) that comprise GEA, the specific elements within these dimensions, and how these elements permeate the SSRL process. This requires the preliminary establishment of a conceptual framework for GEA grounded in existing theories of emotion, emotion awareness, emotion regulation, and SSRL.

*RQ2*: How is Group Emotion Awareness embedded into the collaborative process?

To realize GEA from an SSRL perspective, it must be integrated into the collaborative process. Therefore, it is necessary to construct a corresponding process model that allows GEA to be sustained throughout the entire shared regulation lifecycle.

*RQ3*: How can the Group Emotion Awareness model be operationally applied in collaborative scenarios?

To implement GEA in authentic collaborative tasks, it is essential—under the guidance of the conceptual framework and process model—to clarify how emotional cues are externalized to form shareable information within a team, how shared emotional interpretations evolve into shared representations of emotional attribution problems, and how these further trigger regulatory activities such as planning, monitoring, reflection, and strategy adjustment. This will lead to the identification of reusable instructional implementation essentials and applicable boundaries.

## 3 Construction of the group emotion awareness model

This chapter addresses RQ1 and RQ2 through a progressive model-building process. Section 3.1 establishes the individual-level theoretical foundation of emotion awareness, which serves as the starting point for extending emotion awareness to collaborative settings. Section 3.2 then examines emotion awareness from the perspective of socially shared regulation, explaining the transition from individual to group-level awareness. Building on these foundations, Section 3.3 constructs the group emotion awareness (GEA) conceptual framework and process model and further proposes how the model can be operationalized through instructional scaffolding.

### 3.1 Individual emotion awareness model

Many emotion awareness tools, while providing learners with perceptions of their emotions, often lack a robust theoretical framework to explain how these emotions are elicited within learning contexts or how they can be further transformed into actionable objects of regulation. Consequently, emotion awareness frequently remains at the level of “state presentation,” making it difficult to support subsequent regulated learning (Leony et al., 2013). A comprehensive theoretical model for emotion awareness should, therefore, not only cover the identification of emotions themselves but also incorporate dimensions such as emotional causality and meaningful interpretation. This support allows learners to conduct attributional inquiries into emotional cues and trigger appropriate regulation. Following this rationale, Lavoué et al. (2020) proposed a three-dimensional theoretical framework for emotion awareness that supports emotion regulation, grounded in Pekrun’s Control-Value Theory of Achievement Emotions. This framework provides a more mechanism-oriented and structured pathway for the “perception–interpretation–regulation” cycle.

The present study employs the aforementioned framework by Lavoué et al. for research design and analysis, primarily based on two considerations. First, the framework incorporates the interpretation and attribution of emotional cues into the awareness process with clear, operationalizable dimensions, allowing it to be embedded across different stages of collaborative learning to facilitate subsequent regulation. Second, the framework maintains high consistency with Pekrun’s Control-Value Theory in academic settings, providing a solid theoretical foundation for extending emotion awareness from the individual to the group level. To facilitate its application in more generalized collaborative learning environments, this study further adapts the framework into a “cuboid” structure, forming a conceptual framework for individual emotion awareness (see Figure 1).

**Figure 1:**
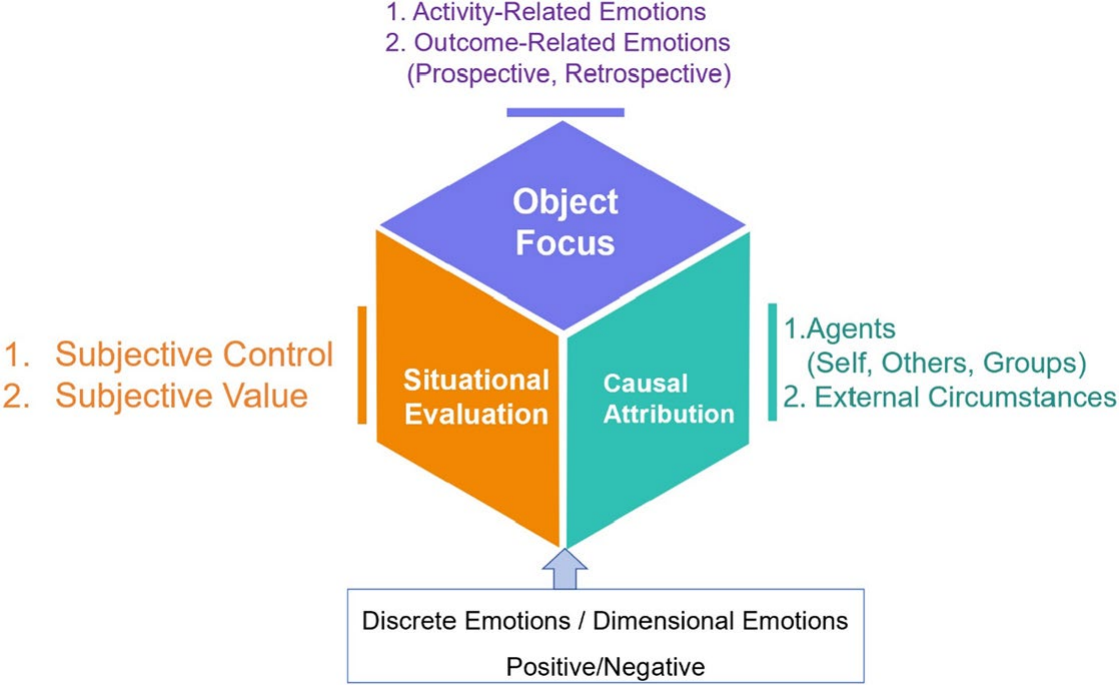
Conceptual framework for individual emotion awareness developed by the authors based on Lavoué et al. (2020) and Pekrun’s Control-Value Theory.

The conceptual framework illustrated above indicates that learners are more likely to select and implement appropriate self-regulation strategies only when they can clarify the object focus of their emotions, assess situation-specific value and control, and make attributional judgments regarding emotional causes. This provides a clear direction for subsequent regulated learning. A prerequisite for this three-dimensional attributional inquiry is the learners’ ability to identify their own emotional states in a timely manner during the collaborative process. Technological tools serve as an effective means to achieve this, reducing the “cost” of emotion recognition and retrospection while enhancing the visibility and traceability of emotional cues. Existing emotion awareness tools predominantly utilize discrete emotion classification systems (basic emotions) or dimensional models (e.g., valence and arousal) to represent learners’ emotional states (Ez-zaouia et al., 2020). Consequently, establishing a stable perception of one’s emotional state based on these classification systems is a fundamental prerequisite for conducting further attributional inquiries and triggering regulatory actions.

### 3.2 Emotion awareness from the perspective of socially shared regulation

Coordination among team members is the cornerstone of effective teamwork (Salas et al., 2005). However, successful coordination does not always occur spontaneously, necessitating regulation across various dimensions of collaborative activity. Theories of socially shared regulation provide a robust framework for research and practice in collaborative learning (Chen et al., 2019). Within the context of computer-supported collaborative learning (CSCL), while emotion awareness is a fundamental prerequisite for emotion regulation (Boden and Thompson, 2015), its catalytic effects during the collaborative process extend far beyond the purely emotional layer.

#### 3.2.1 Emotion awareness permeates all stages of socially shared regulation

Emotion awareness activities within the framework of shared regulation allow group members to establish a firm socio-emotional foundation, upon which the collective can execute corresponding tasks (Miele, 2009; Boekaerts and Pekrun, 2015). Sustained collaboration is only guaranteed when members’ emotions and affects are subject to shared monitoring and regulation throughout the learning process. Conversely, low-quality monitoring and regulation can lead to adverse emotions (e.g., anxiety and dissatisfaction) and extend negative impacts to subsequent stages of collaboration (Kwon et al., 2014). Research by Vega (2014) investigated learners’ actions in response to academic and social challenges, finding that emotion awareness significantly influences students’ shared regulated learning, group engagement, and collaboration. When collaborative groups face academic hurdles, they adopt adaptive regulatory strategies to remain task-focused and mitigate negative emotions, thereby manifesting socially shared regulatory behaviors. In an in-depth study of two cases of shared regulation, Ucan and Webb (2015) found that the shared regulation of emotions and affects during cognitive processes sustains student interactions. A prerequisite for such regulation is the gradual externalization of a learner’s implicit affect into a conscious state. As both a condition and a product of socially shared regulation, emotion serves as a foundation that influences group monitoring, reflection, and evaluation; meanwhile, shared monitoring and evaluation during collaboration, in turn, create and maintain positive emotional states.

#### 3.2.2 Emotion awareness acts on all dimensions of socially shared regulation

Emotion represents a reaction to specific situations (Rosenberg, 1998) based on multidimensional psychological processes, including affective, cognitive, physiological, motivational, social, and expressive components (Russell and Barrett, 1999; Pekrun et al., 2011). In collaborative learning environments, when students perceive emotions, they may directly regulate their individual-level affect (Bakhtiar et al., 2018). Furthermore, when students reciprocally perceive each other’s emotions within a group, the regulatory strategies employed may vary depending on the task domain. They utilize cognitive, motivational, behavioral, and interpersonal strategies to varying degrees (Lobczowski, 2019). For instance, if emotion awareness reveals that anxiety is attributed to cognitive difficulties, the group may employ cognitive strategies to adjust their goal setting. Individuals or groups can also use motivational strategies to regulate emotions; for example, two emotionally conflicted students might compare their relationships with each other against their relationships with other peers to enhance their motivation to cooperate. Behavioral strategies involve altering actions to regulate emotions—such as a learner taking a walk to release stress when worried about achieving collaborative goals. Finally, interpersonal strategies are driven by one’s relationships with others (Van Kleef, 2016), such as sharing the joy of achievement or venting to others to alleviate sadness when feeling low.

#### 3.2.3 Emphasis on the transition from individual to group emotion awareness

While emotion regulation in individual learning contexts is well-established (Folkman and Lazarus, 1988; Gross, 2015), research in collaborative settings remains largely confined to the individual level. In recent years, scholars have begun to examine emotion regulation through the lens of shared regulation, defining research in the emotional dimension of socially shared regulation of learning (SSRL) as Socially Shared Emotion Regulation (Kazemitabar et al., 2022). This concept spans the trajectory from emotional self-regulation to co-regulation and ultimately to socially shared emotion regulation. According to Gross’s (2015) definition, emotion regulation is “the process by which individuals influence which emotions they have, when they have them, and how they experience and express these emotions.” Clearly, traditional research is rooted in the individual level. The expansion of Gross’s model by Kazemitabar et al. (2022) led to the formation of the group-level socially shared emotion regulation model, representing an extension of emotion regulation theory from a single individual dimension to a collective dimension.

Similarly, shared regulation across cognitive, metacognitive, motivational, and behavioral levels is also driven by the migration of emotion awareness to the group level. As regulation transitions from the individual to the group, complexity increases because each student contributes unique regulatory skills and knowledge to the collective (Järvenoja et al., 2013). In this process, emotion awareness serves as the primary driver of members’ regulatory behaviors. Members take action based on their understanding of both personal and collective emotional states, employing shared regulation strategies during interactions. These actions affect regulatory content across the aforementioned levels, achieving adjustment and adaptation within individual, peer, and collective scopes. To better explain and analyze this cyclical and iterative “perception-regulation” process, this study constructs the Group Emotion Awareness model described in the following section.

### 3.3 The group emotion awareness (GEA) model

#### 3.3.1 Establishment of the conceptual framework

Drawing upon Pekrun’s Control-Value Theory of Achievement Emotions, as described in our previous individual emotion model, when individual learners perceive their emotions during the learning process, this awareness elevates to an exploration of the underlying causes, thereby forming corresponding self-regulation strategies. When emotion awareness migrates from the individual to the group level, individuals or groups explore the causes of emotional shifts and states based on a synthesized perception of the internal emotional distribution within the team. Subsequently, they engage in socially shared regulation across multiple dimensions, including cognition, metacognition, affect, motivation, and behavior. Given the mutually interactive and interdependent nature of Group Emotion Awareness (GEA) and Socially Shared Regulation of Learning (SSRL), this study develops a conceptual framework for Group Emotion Awareness (see Figure 2). This framework is built upon the previously established individual emotion awareness framework and incorporates Kazemitabar’s transition process from individual to group in socially shared emotion regulation. It integrates GEA into the dynamic activities of SSRL, achieving emotional perception without altering the original collaborative process or increasing collaborative and cognitive load.

**Figure 2:**
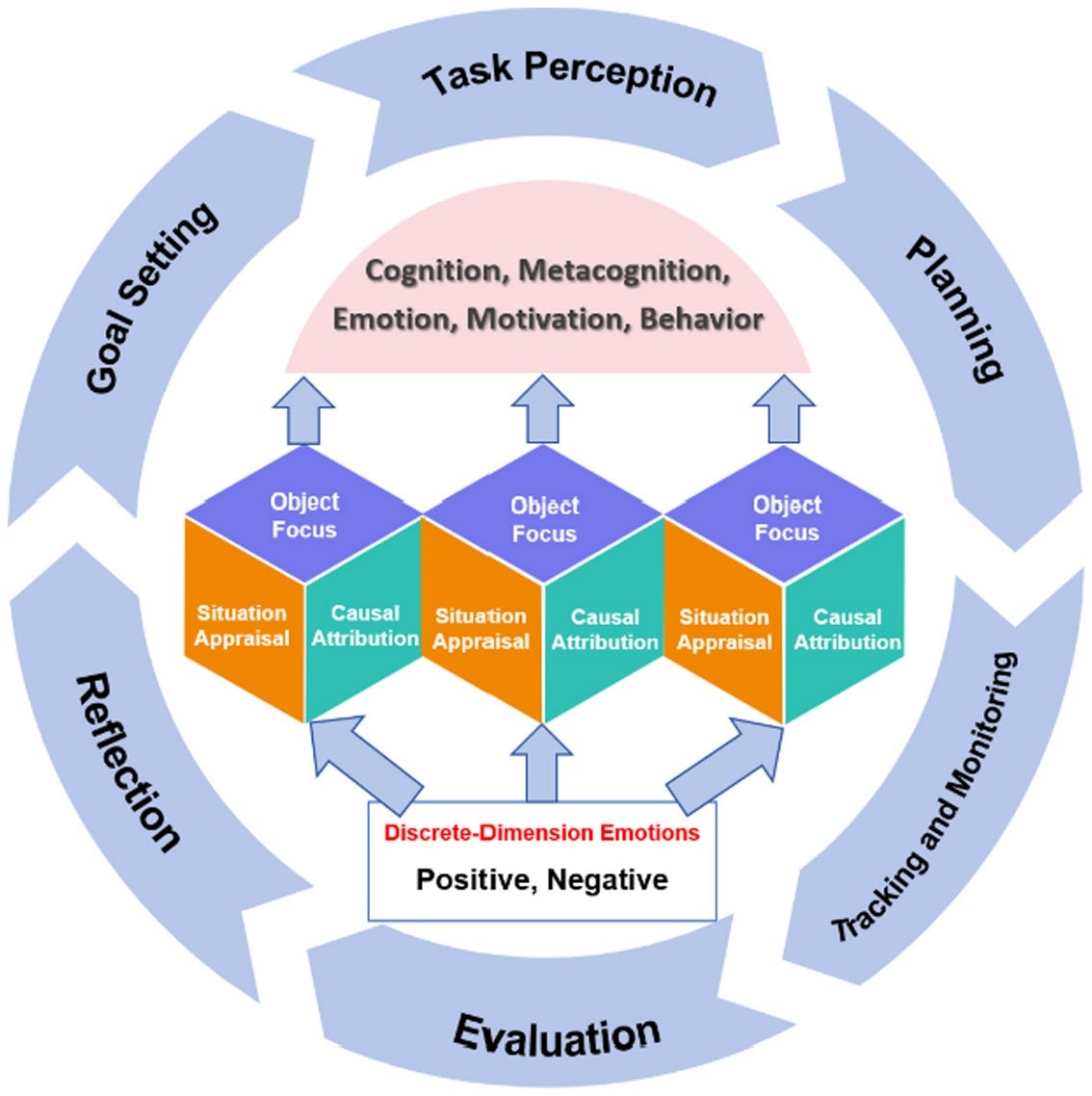
Conceptual framework for group emotion awareness.

To clarify the distinction between model proposal and evidence support, this study summarizes the functional mechanisms of GEA within SSRL into three propositions:

(P1) Self-emotion awareness triggers a three-dimensional inquiry—focusing on object focus, situational appraisal, and causal attribution—and primarily operates at the individual level of regulation.
(P2) Supported by tools, communication, and scaffolding, individual perceptions of peers’ emotions expand into shared information, fostering shared interpretation and elevating specific causes into shared problem representations.
(P3) Based on these shared representations, teams trigger and transition between self-regulated learning (SRL), co-regulated learning (CoRL), and SSRL regulatory modes, thereby influencing the quality of collaborative processes and learning outcomes.

These propositions are not purely theoretical assumptions; rather, they are abstracted from recurring patterns identified in prior empirical research on emotion, regulation, and collaborative learning, and are further examined through the theory-driven evidence-chain synthesis presented in Section 4.

#### 3.3.2 Establishment of the process model

To operationalize the conceptual framework within authentic collaborative settings, this study constructs a process model for group emotion awareness. The design of this process model follows three core principles:

1. Full-cycle Embedding: Integrating emotion awareness into the start, during, and end phases of shared regulation.
2. Linkage with Shared Regulation: Aligning emotion awareness with subsequent regulatory activities, such as planning, monitoring, reflection, and strategy adjustment.
3. Emphasis on Visualization: Presenting emotional states and related cues intuitively to reduce perception costs and support shared understanding.

Numerous researchers have explored the application of group awareness and corresponding tools in collaborative learning, developing various application modes (Sheng et al., 2019; Zhang et al., 2023). Our research team previously established the “Group Awareness Application Framework for Shared Regulation” (Chen et al., 2021). By synthesizing the requirements of emotion awareness regarding collaborative processes, awareness content, and formats, this study proposes the GEA process model as shown in Figure 3.

**Figure 3:**
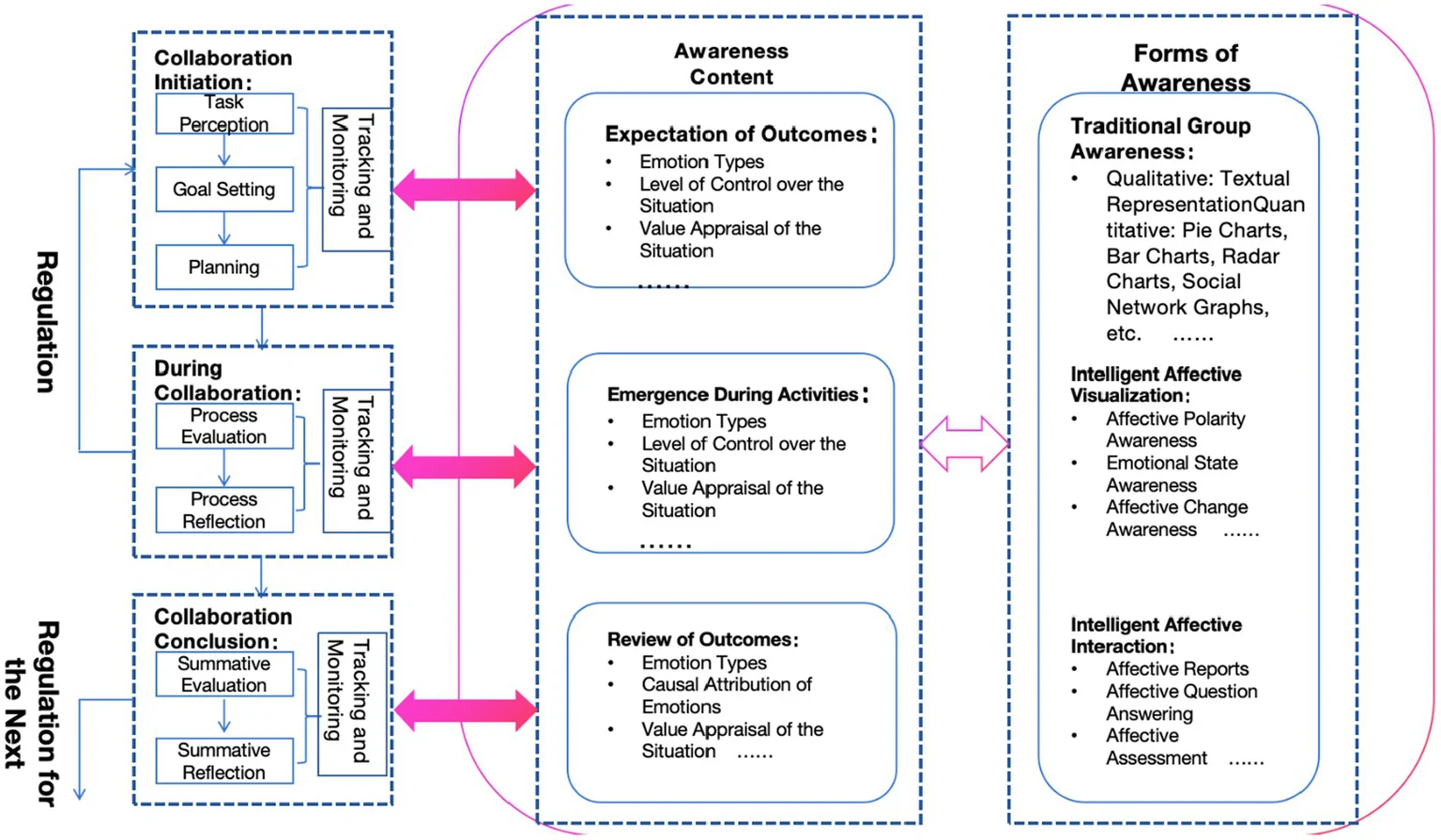
Process model for group emotion awareness.

As illustrated in Figure 3, the GEA process model deeply embeds emotion awareness into the three core stages of SSRL(Start, Process, and End). The left side of the model outlines conventional regulatory activities—from task planning and process reflection to summative evaluation. Through a “monitoring” mechanism, these activities interact dynamically with the awareness module on the right. The awareness module consists of two components: Awareness Content and Awareness Format. Awareness Content not only tracks emotional types across different stages but also delves into deep structures such as situational control, value appraisal, and causal attribution. Awareness Format balances traditional qualitative and quantitative charts (e.g., text, radar charts) while innovatively incorporating emotional visualization and interaction functions powered by cutting-edge intelligent technologies (e.g., Large Language Models). This design aims to make elusive and implicit emotions tangible, thereby providing comprehensive support for the team’s shared regulation.

#### 3.3.3 Operationalization of the process model through instructional scaffolding

This section describes how the proposed process model can be operationalized through two types of instructional scaffolding. These scaffolds have been applied in our related empirical work; however, the empirical implementation and evaluation are reported separately and are therefore beyond the scope of this conceptual paper. In instructional settings, operationalizing the process model requires translating emotion awareness, emotional attribution, and shared regulation into actionable instructional scaffolding. Accordingly, the operationalization of the model involves two complementary types of scaffolding: Emotion Awareness Measurement Scaffolding and Post-Perception Regulatory Scaffolding (see Figure 4).

**Figure 4:**
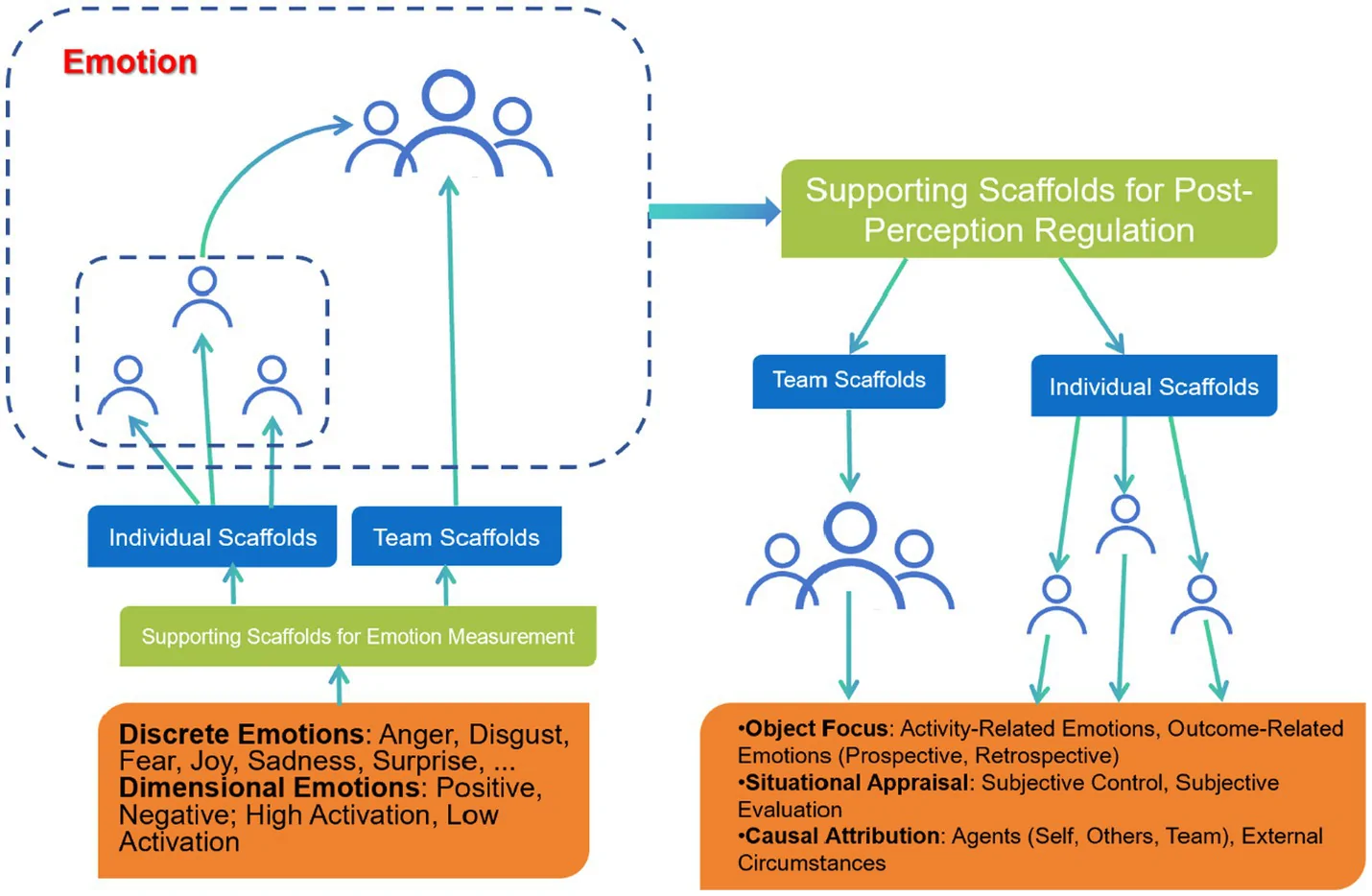
The scaffolding process supporting group emotion awareness.

Emotion Awareness Measurement Scaffolding assists individuals and teams in timely externalizing and aggregating emotional information during collaboration. Post-Perception Regulatory Scaffolding guides members in conducting interpretation and attribution based on emotional cues, thereby triggering regulatory activities such as planning, monitoring, reflection, help-seeking, and strategy adjustment.

As shown in Figure 4, these two types of scaffolding correspond to the “externalization and sharing of emotional cues” and “interpretation-based regulatory behavior,” respectively, thereby embedding group emotion awareness into the socially shared regulation of learning collaborative process. Section 4 will provide further evidence support for this mechanistic chain through representative empirical research.

## 4 Functional mechanisms and representative empirical research of group emotion awareness

This chapter outlines the implementation paths and applicable boundaries of Group Emotion Awareness (GEA) in collaborative scenarios, grounded in the mechanistic propositions (P1–P3) and synthesized through a theory-driven evidence-chain synthesis approach that integrates representative empirical studies to support mechanism-oriented explanations rather than providing an exhaustive or systematic review of the literature. This approach prioritizes explanatory coherence over exhaustive coverage and aligns empirical evidence with theoretically defined mechanisms. The following evidence synthesis is organized around the three propositions proposed in the previous section.

Accordingly, the empirical studies reviewed in this chapter are intentionally presented after the model construction, as their primary purpose is not to establish the theoretical background from which the model was derived, but to examine the extent to which existing empirical findings provide support for the mechanisms articulated in Propositions P1–P3.

### 4.1 Functional mechanisms

#### 4.1.1 Individual emotion awareness and three-dimensional attributional inquiry

Emotion awareness is not merely the identification of a single emotional state; rather, it functions as a process of problem representation signaled by emotional changes. Specifically, it involves how individuals consciously perceive the emotional processes they experience during learning. Emotions are not passive reactions but cognitive inputs and meaningful signals regarding the current situation (Schwarz and Clore, 1983; Clore and Huntsinger, 2007). Through immediate inferences—such as “Why do I feel this way?”—emotions guide individuals to re-appraise task contexts and reconstruct problem representations (Clore and Huntsinger, 2007; Harley et al., 2019). In learning contexts, achievement emotions are posited to originate from learners’ appraisals of situational control and task value (Pekrun, 2006; Pekrun and Perry, 2014); thus, emotional volatility itself reflects a dynamic understanding of the problem structure.

When learners experience confusion, anxiety, frustration, or excitement during collaborative tasks, these emotional experiences trigger a structured inquiry into the sources of emotion. This inquiry can be categorized into three dimensions: object focus, situational appraisal, and causal attribution. First, emotions can be directed toward learning activities or outcomes, distinguishing between “activity emotions” and “outcome emotions” (Pekrun et al., 2002). Second, emotions arise from subjective assessments of control and task value, constituting the situational appraisal dimension (Pekrun et al., 2006; Pekrun and Perry, 2014). Third, learners make attributional judgments regarding emotional origins—such as attributing failure to internal lack of ability or external environmental constraints—which subsequently influence behavioral tendencies (Weiner, 1985; Wang and Eccles, 2013). Consequently, the “object focus—situational appraisal—causal attribution” triad constitutes an emotion-driven framework for problem re-representation.

This three-dimensional inquiry process does not remain at an abstract reflective level but manifests in specific choices of regulatory behavior. According to Self-Regulated Learning (SRL) theory, regulation can be divided into cognitive, metacognitive, motivational, and emotional dimensions (Zimmerman, 2000; Boekaerts, 2011; Tinajero Vacas et al., 2024). When the object focus points toward a “knowledge gap,” learners are more likely to adopt cognitive strategies (e.g., re-reading materials, intensive practice). When appraisal indicates “insufficient task value,” motivational or emotional regulation (e.g., enhancing interest or self-encouragement) is more likely to be activated. When attribution points to external situational factors (e.g., time constraints or task standards), it triggers metacognitive monitoring and planning adjustments. Attribution theory further suggests that internal-controllable attributions are more likely to stimulate effort and strategy use, whereas external-uncontrollable attributions may undermine sustained engagement (Weiner, 1985).

Through the individual-level “three-dimensional inquiry” mechanism, group emotion awareness (GEA) transforms implicit emotional experiences into actionable objects of regulation. Learners represent problems as “knowledge gaps,” “value deficits,” or “situational constraints,” choosing different regulations across cognitive, metacognitive, motivational, emotional, or behavioral dimensions. This process validates Proposition P1: emotion awareness is essentially a dynamic mechanism that uses emotional change as a signal to drive the generation and reconstruction of problem representations.

#### 4.1.2 Sharing emotional information: from individual perception to group interpretation

In collaborative settings, learners’ emotion awareness expands from the “self” to “others” and, supported by tools, communication, and scaffolding, is further transformed into team-level shareable information. This implies that emotional information in collaboration does not remain isolated within individual experience but is externalized, noticed, and discussed through interaction, ultimately becoming part of the team’s collective understanding (Rimé, 2009; Rimé et al., 2020). Research demonstrates that emotions can be captured, visualized, and integrated into team regulatory processes through technological tools.

Multiple studies have shown that AI and multimodal analysis technologies allow implicit emotional states to become identifiable team resources. For instance, facial-recognition-based affect analysis reveals individual emotional states and their dynamic correlations within groups, bringing emotions to an observable and analyzable level (Ngo et al., 2024). Furthermore, by utilizing Hidden Markov Models to analyze emotional transition patterns during collaboration, research has uncovered the dynamic structure of how emotions trigger regulatory behaviors in challenging scenarios (Dang et al., 2023). Cross-modal analysis (facial expressions and discourse interaction) further shows a co-occurrence between emotions and socially shared regulation behaviors, particularly in expert teams where emotion and socio-cognitive interaction are highly coupled (Huang et al., 2025). Simultaneously, multimodal framework research indicates that “shared emotional shifts” (emotional synchrony) often accompany the construction of shared understanding (Aoyama Lawrence and Weinberger, 2022). These studies collectively suggest that technological tools enhance the “visibility” of emotional cues, transforming them from internal experiences into signals within the team’s attentional resources.

Second, scaffolding and regulatory scripts have been shown to facilitate the naming, interpretation, and negotiation of emotions, thereby forming shared understanding. Regulation macro-scripts provide teams with structured reflection phases, allowing members to externalize emotions (e.g., frustration) and attribute them to specific task difficulties (Näykki et al., 2021). The technology-supported “6Q” tool enhances students’ awareness of motivational and emotional states, triggering group-level regulatory discussions (Järvenoja et al., 2020). AI-driven learning analytics research further found that emotion-triggering events often initiate negotiative interactions within groups, forming distinct regulatory path patterns (e.g., “plan-act” vs. “trial-and-error”), suggesting that teams filter and integrate the problems signaled by emotions (Dang et al., 2024). In online collaborative environments, negative social emotions are frequently triggered by goal misalignment; notably, socially shared emotion regulation is significantly negatively correlated with negative emotions, highlighting the critical role of shared regulation in aligning emotional interpretations (Liu and Ding, 2025). These findings reveal that emotional cues only transform into team-level shared understanding after being named, discussed, and proposed as candidate interpretations.

Finally, research shows that emotional signals can be reconstructed as shared problem objects for the team, thereby triggering socially shared regulation. The Human-AI Shared Regulation of Learning model proposes that AI can identify regulatory triggers and support teams in transforming individual emotions into shared regulatory goals (Järvelä et al., 2023). AI-generated multimodal timelines further reveal “difficulty thresholds” and “intervention points,” providing a basis for teams or teachers to identify when to elevate individual difficulties to team-level issues (Cohn et al., 2025). Meanwhile, comparative studies of expert and novice teams show that experts are better at integrating high-arousal emotions with socio-cognitive interactions to form coordinated regulatory patterns, whereas novices struggle with this “causal elevation” process (Huang et al., 2025). This indicates that the transformation of emotions from “individual signals” to “team problem representations” is a vital prerequisite for the occurrence of socially shared regulation.

Through technological tools, emotional cues are externalized and entered into team attentional resources, forming shared interpretations through naming and negotiation, and ultimately elevating to team-level problem representations. This continuous process provides objects and direction for socially shared regulation, validating Proposition P2 regarding the dynamic transformation path of emotion from the individual to the team level.

#### 4.1.3 Regulatory mode switching: SRL, CoRL, SSRL and its impact

Teams do not remain in a single regulatory mode throughout collaboration but trigger and switch between Self-Regulated Learning (SRL), Co-Regulated Learning (CoRL), and Socially Shared Regulation of Learning (SSRL) based on varying contexts and needs (Järvelä et al., 2015; Hadwin et al., 2017). Emotion awareness acts as a “threshold signal” in this process: when emotional cues suggest risk accumulation, goal obstruction, or member divergence, the team is more likely to shift from individual-level regulation to interactive or shared regulation (Törmänen et al., 2021; Mänty et al., 2023). Research indicates that these switches are often triggered by emotional and motivational signals and are closely related to task progress, interaction structures, and emotional consistency.

A growing body of research suggests that emotional and motivational challenges often function as “trigger events” that prompt regulation to shift from individual to interactive or shared levels. When motivational difficulties or emotional disturbances occur, CoRL is dynamically activated under situational challenges, supporting the generation and development of SRL and SSRL (Bakhtiar and Hadwin, 2020). In the presence of negative social emotions, teams tend to adopt socially shared emotion regulation, which significantly inhibits negative affect (Liu and Ding, 2025). Concurrently, motivational regulation is often embedded within the broader SSRL process; when motivational and cognitive regulation occur synchronously, individuals perceive stronger peer support and regulatory efficacy (Järvenoja et al., 2025). These studies collectively support the mechanism of emotional cues as threshold signals triggering the escalation of regulatory levels.

Second, the choice of regulatory mode is closely tied to task progress. Regulatory modes must match situational challenges to facilitate knowledge co-construction (Bakhtiar and Hadwin, 2020). High-performance teams typically exhibit complete and structured SSRL phases (task analysis, planning, monitoring, and reflection), which are significantly correlated with higher academic achievement (Xu and Lou, 2024). In complex problem-solving tasks, planning and reflection form a structural framework, while monitoring and reasoning alternate cyclically during execution, presenting a dynamic regulatory path (Jablansky, 2020). This suggests that as task obstruction or complexity increases, the object of regulation is elevated from the individual to the interactive and shared levels.

Third, interaction structures and the degree of responsibility interdependence influence the upgrade of regulatory modes. CoRL plays a key mediating role in supporting SRL and cognitive presence, promoting learning progress through indirect paths (Zheng et al., 2023). Compared to CoRL, SSRL emphasizes joint goal construction, shared monitoring, and collective control, representing a higher-level regulatory mode (Bransen et al., 2022). Empirical research shows that SSRL often emerges during critical moments of increasing engagement, team coordination needs, or situational challenges (Isohätälä et al., 2017). Thus, in contexts of high interdependence and collective decision-making, SSRL is more likely to be triggered.

Additionally, emotional distribution consistency and interpretation alignment affect the formation of SSRL. High-performance teams exhibit a higher proportion of positive activating emotions during SSRL phases, which correlates positively with academic performance (Xu and Lou, 2024). Individuals with strong SRL skills are more inclined to initiate SSRL and positive social emotion regulation (Li et al., 2019). SSRL is typically accompanied by synchronized increases in engagement rather than significant divergence among members (Isohätälä et al., 2017). This indicates that consistency in emotional and cognitive interpretation facilitates the emergence of shared regulation.

Finally, the escalation and switching of regulatory modes can influence collaborative process quality and learning outcomes. SSRL has been associated with higher academic achievement and better team product quality (Panadero and Järvelä, 2015; Xu and Lou, 2024). Recent research using AI and learning analytics has also identified regulatory trigger points and adaptive regulatory paths, including distinct patterns such as “plan-act” and “trial-and-failure” modes (Dang et al., 2024). Technology-supported tools can further facilitate SSRL by externalizing regulatory information and providing prompts for shared regulation (Järvelä et al., 2015, 2023). Taken together, these findings suggest that regulatory mode switching is associated with differences in planning, monitoring, conflict resolution, and strategy adjustment, which may in turn influence collaborative processes and learning outcomes.

Following the emergence of emotional and motivational trigger events, the regulatory mode often upgrades from SRL to CoRL/SSRL, thereby driving changes in collaborative process quality and ultimately reflecting in differences in learning outcomes. This continuous process echoes and supports Proposition P3: teams trigger and switch between SRL, CoRL, and SSRL based on relevant cues, thereby influencing collaborative process quality and learning outcomes.

### 4.2 Representative empirical research

#### 4.2.1 Collaborative task contexts and regulatory demands

Group Emotion Awareness and socially shared regulation do not appear stably in all cooperative contexts; they are typically embedded in collaborative tasks characterized by interdependence, phase-based progress, and joint decision-making needs. Socially shared regulation of learning (SSRL) research consistently emphasizes the multi-level nature of collaborative regulation: self-regulated learning (SRL), co-regulated learning (CoRL), and SSRL can coexist and undergo dynamic transformations. Therefore, research must provide “space” within task structures for these transformations (Järvelä and Hadwin, 2013; Järvelä et al., 2016a; Hadwin et al., 2017).

On one hand, in authentic or quasi-authentic collaborative learning, regulatory activities are not uniformly distributed but are concentrated at critical junctures (e.g., understanding conflicts, insufficient evidence, unbalanced division of labor, time pressure). For example, Janssen et al. (2012) distinguished between task-related and social regulation in online collaborative learning, noting that both unfold in parallel and change dynamically as the task progresses. Correspondingly, Volet et al. (2009) discussed the conditions for emerging and maintaining high-level co-regulation, emphasizing its dependence on continuous goal alignment and shared monitoring. Similarly, Ucan and Webb (2015) revealed how social regulation emerges and functions in collaborative inquiry, suggesting that regulation is not an individual attribute but a product of context and interaction.

On the other hand, when teams struggle to establish a joint problem space or effectively coordinate differences, even highly capable individuals may fall into inefficient interactions, forcing regulatory needs to manifest passively amidst conflict and stagnation (Barron, 2003). More recent intervention studies show that promoting SSRL through scaffolding in online project-based courses significantly impacts team collaboration quality and output, demonstrating that the “regulatory demand—regulatory behavior—output quality” chain is interventional in authentic task contexts (Cortázar et al., 2022).

#### 4.2.2 Emotional cues and externalization

This subsection focuses on how emotional cues are captured and integrated into shared information systems. Learning science and computer-supported collaborative learning (CSCL) research generally agree that in collaborative settings, emotions do not automatically become shared objects; they often present as fragmented signals in language, behavior, and interaction structures. Only when these signals are externalized, visualized, or guided by scripts into a common focus can emotional information be further interpreted, aligned, and utilized for regulation. Group awareness research mechanically explains this: awareness tools provide shareable information about member status, participation structures, or learning activities, helping teams identify differences and risks (Bodemer and Dehler, 2011; Janssen and Bodemer, 2013; Bodemer et al., 2018).

In the CSCL context, scripts provide directional focus, interaction rules, and structural task requirements, while awareness tools provide referable evidence of status, thereby altering how teams monitor and regulate (Fischer et al., 2013; Miller and Hadwin, 2015). Emotional awareness research specifies “visibility” through emotion visualization or dashboards: Leony et al. (2013) demonstrated paths for providing emotional awareness via visualization in computer-mediated environments; Ez-Zaouia et al. (2020) proposed *EMODASH* to support retrospective awareness of emotional experiences in online learning; Feidakis et al. provided emotional awareness and feedback in virtual collaborative settings, emphasizing that emotional information is presentable and feedback-capable within collaborative systems (Feidakis et al., 2013, 2014a).

Lavoué et al. (2020) explicitly argued that emotion awareness tools should serve emotion and appraisal regulation, advancing emotional information from “state presentation” to “interpretable and re-appraisable” learning support. In research closer to CSCL instructional practice, group awareness tools have been shown to influence online collaborative writing, knowledge construction, learning engagement, and performance, providing empirical support for the idea that “externalization brings measurable change” (Phielix et al., 2011; Liu et al., 2018; Li et al., 2021; Peng et al., 2022).

#### 4.2.3 Emotional interpretation and problem representation

Emotional cues transform into regulatory objects only after being interpreted and integrated into shared understanding. Socio-emotional processes in collaboration are intertwined with cognitive progress. Emotions influence participation styles and argumentation quality, while also altering the team’s meaning-making of current difficulties through interaction rhythms and relationship maintenance. Näykki et al. (2014) presented the processual characteristics of socio-emotional conflict in higher education; Isohätälä et al. (2018, 2020) further revealed how socio-emotional processes fluctuate with participation in collaborative argumentation, co-shaping the trajectory of collaborative progress alongside cognitive interaction.

Evidence regarding “causal elevation/shared problem representation” comes from socially shared metacognition research. Hurme et al. (2009) found in computer-supported collaborative learning (CSCL) mathematics courses for teacher education that members’ feelings of “task difficulty” can be shared and discussed in interaction, elevating to collective metacognitive objects that influence team monitoring and regulation. Additionally, regulatory scripts can be designed to promote both socio-cognitive and socio-emotional monitoring: Näykki et al.’s (2017) research on macro-scripts showed that scripted prompts can encourage teams to shift attention from individual experiences to shared monitoring objects, providing interactional conditions for shared interpretation.

The mechanistic expression from “emotional interpretation” to “team-level regulatory objects” can be assisted by team emotion regulation process models. Kazemitabar et al. (2022), expanding Gross’s individual model, proposed a team emotion regulation process model emphasizing a chain of identifying, interpreting, and coordinating actions based on emotional cues. Bakhtiar et al. (2018) compared regulation and socio-emotional interaction characteristics under different group climates, providing process evidence for how “interpretation and climate” affect subsequent regulation.

#### 4.2.4 Regulatory process indicators and mode differentiation

After emotional cues are interpreted and shared problem representations are formed, researchers observe whether identifiable regulatory process indicators emerge to differentiate and infer self-regulated learning (SRL), co-regulated learning (CoRL), and socially shared regulation of learning (SSRL) modes and their transition paths. SSRL theory explicitly characterizes collaborative regulation across two dimensions: “regulatory agent” and “regulatory object.” SRL is primarily initiated by the individual and directed toward individual tasks; CoRL involves peer guidance, prompting, and support, though the object may remain local; SSRL centers on shared goals and shared problem representations, with monitoring and decision-making being collective and interdependent (Järvelä and Hadwin, 2013; Järvelä et al., 2016a; Hadwin et al., 2017).

Järvelä et al. (2015, 2016b) proposed designing computer-supported collaborative learning (CSCL) regulation tools to promote SSRL, emphasizing process indicators like joint planning, shared monitoring, and collective reflection. Furthermore, Järvelä et al. (2016a) used time-series analysis of online chats and logs to identify SSRL, demonstrating that regulatory activities possess identifiable temporal structures. Building on this, Malmberg et al. (2015, 2017) revealed differences in the sequential patterns of regulation between high- and low-performance teams by capturing SRL, CoRL, and SSRL modes, providing a fine-grained analytical path for “mode differentiation and transformation.” Complementing sequential analysis is process mining: Schoor and Bannert (2012) demonstrated how to use process mining to identify regulatory processes in computer-supported tasks, providing a methodological reference for replicable analysis.

Janssen et al. (2012) showed that task-related and social regulation occur in parallel, suggesting that teams are more likely to shift from individual coping to interactive coordination as social-dimensional risks accumulate. Sobocinski et al. (2020) further revealed how monitoring “sets the stage” for subsequent adaptive regulation or maladaptive behavior, emphasizing that regulation is not inherently effective but depends on its sequential structure and timing. Additionally, when teams have misaligned task requirements or division-of-labor expectations, collaboration faces systemic challenges; script-based interventions in shared task perception can alter team representations, thereby influencing regulatory activities and collaborative outcomes (Hadwin et al., 2018). At the intervention level, Cortázar et al. (2022) also provided empirical support for the impact of SSRL scaffolding on online project-based team collaboration and outcomes.

## 5 Conclusion and discussion

The key contribution of this study is to conceptualize group emotion awareness not merely as emotional visibility, but as a mechanism through which emotional cues are interpreted, attributed, and transformed into shared objects of regulation.

From the perspective of SSRL, this study proposes conceptual and process models of Group Emotion Awareness (GEA). First, grounded in Control-Value Theory and a three-dimensional framework of emotion awareness, the conceptual model explains how emotional awareness extends from emotion perception to causal inquiry, shared interpretation, and shared problem representation at the group level. Second, the process model embeds emotion awareness across collaborative phases through emotion-awareness and post-perception regulatory scaffolding, supporting the transformation of emotional cues into shared regulation.

To distinguish between model proposal and evidence support, the functional mechanism of GEA within SSRL is summarized into three propositions: (P1) individual emotion awareness triggers inquiry into emotional causes and supports individual regulation; (P2) emotional information is shared and interpreted through tools and scaffolding, allowing emotional causes to develop into shared problem representations; and (P3) teams dynamically shift between SRL, CoRL, and SSRL, thereby influencing collaborative processes and learning outcomes. Based on these propositions, Section 4 adopts a theory-driven evidence-chain synthesis to explain how emotional cues are externalized, interpreted, and transformed into shared regulatory objects in collaborative learning. The model further embeds emotion awareness into SSRL processes through scaffolding and process-level support, linking theoretical understanding with instructional implementation.

This study extends existing computer-supported collaborative learning (CSCL) research on emotion awareness by arguing that emotional externalization is not an endpoint but a starting point for interpretation and regulation. Rather than merely visualizing emotions, the proposed model emphasizes how emotional cues trigger attributional inquiry, shared interpretation, and shared problem representation, shifting the focus from “seeing emotions” to using emotions as signals for regulation. Consistent with the SSRL perspective, emotion awareness is further conceptualized as a threshold signal that supports transitions between SRL, CoRL, and SSRL through activities such as planning, monitoring, and reflection. By embedding emotion awareness into the collaborative process through scaffolding and process-level support, the model transforms emotion from a background factor into an actionable mechanism for shared regulation. The theoretical contribution of this study therefore lies in providing a more mechanistic and actionable framework for GEA to support future tool design and instructional implementation.

Several limitations should be noted. First, as a theory-driven evidence-chain synthesis rather than a systematic review or meta-analysis, this study emphasizes mechanistic explanation over exhaustive evidence coverage or quantitative synthesis. Second, variations in collaborative tasks, emotional data sources, and regulatory indicators limit cross-study comparability, highlighting the need for more standardized emotional and SSRL process measures in future research. Third, although prior studies support emotional externalization and shared regulation, direct evidence regarding key mediating processes—particularly the formation of shared interpretations and shared problem representations—remains limited. Future studies should therefore employ more process-oriented methods, such as conversation analysis, sequence analysis, and process mining, to further validate the proposed mechanisms. Finally, although this paper does not present new empirical data, the proposed framework is grounded in the authors’ ongoing empirical research on scaffolding-supported GEA, including a published empirical study on emotional cue externalization (Chen et al., 2024) and continuing work on emotion regulation scaffolding and attributional support.

Future research should address these limitations through continued implementation studies. More transparent evidence reporting and consistent indicators across collaborative contexts may improve the comparability and accumulation of evidence regarding emotional cue externalization, shared interpretation, shared problem representation, and regulatory mode switching. In addition, more process-oriented evidence is needed to examine key mediating mechanisms and support the iterative refinement of scaffolding and the GEA model across collaborative settings.

## Data Availability

The original contributions presented in the study are included in the article/supplementary material, further inquiries can be directed to the corresponding author.

## References

[ref1] Akçay M. B. Oğuz K. (2020). Speech emotion recognition: emotional models, databases, features, preprocessing methods, supporting modalities, and classifiers. Speech Comm. 116, 56–76. doi: 10.1016/j.specom.2019.12.001

[ref2] Aoyama Lawrence L. Weinberger A. (2022). Being in-sync: a multimodal framework on the emotional and cognitive synchronization of collaborative learners. Front. Educ. 7:867186. doi: 10.3389/feduc.2022.867186

[ref3] Bakhtiar A. Hadwin A. F. (2020). Dynamic interplay between modes of regulation during motivationally challenging episodes in collaboration. Front. Learn. Res. 8, 1–34. doi: 10.14786/flr.v8i2.561

[ref4] Bakhtiar A. Webster E. A. Hadwin A. F. (2018). Regulation and socio-emotional interactions in a positive and a negative group climate. Metacogn. Learn. 13, 57–90. doi: 10.1007/s11409-017-9178-x

[ref5] Barrett L. F. Gross J. Christensen T. C. Benvenuto M. (2001). Knowing what you’re feeling and knowing what to do about it: mapping the relation between emotion differentiation and emotion regulation. Cogn. Emot. 15, 713–724. doi: 10.1080/02699930143000239

[ref6] Barron B. (2003). When smart groups fail. J. Learn. Sci. 12, 307–359. doi: 10.1207/S15327809JLS1203_1

[ref7] Bodemer D. Dehler J. (2011). Group awareness in CSCL environments. Comput. Hum. Behav. 27, 1043–1045. doi: 10.1016/j.chb.2010.07.014

[ref8] Bodemer D. Janssen J. Schnaubert L. (2018). “Group awareness tools for computer-supported collaborative learning,” in International Handbook of the Learning Sciences, eds. Frank Fischer, Cindy E. Hmelo-Silver, Susan R. Goldman, and Peter Reimann (New York, NY: Routledge), 351–358.

[ref9] Boden M. T. Thompson R. J. (2015). Facets of emotional awareness and associations with emotion regulation and depression. Emotion 15, 399–410. doi: 10.1037/emo0000057, 25706832

[ref10] Boekaerts M. (2011). “Emotions, emotion regulation, and self-regulation of learning: Center for the Study of learning and instruction, Leiden University, the Netherlands, and KU Leuven,” in Handbook of Self-Regulation of Learning and Performance, eds. Barry J. Zimmerman and Dale H. Schunk (New York, NY: Routledge).

[ref11] Boekaerts M. Pekrun R. (2015). “Emotions and emotion regulation in academic settings,” in Handbook of Educational Psychology, eds. Lyn Corno and Eric M. Anderman (New York, NY: Routledge), 90–104.

[ref12] Brackett M. A. Rivers S. E. (2014). “Transforming students’ lives with social and emotional learning,” in International Handbook of Emotions in Education, eds. Reinhard Pekrun and Lisa Linnenbrink-Garcia (New York, NY: Routledge).

[ref13] Bransen D. Govaerts M. J. B. Panadero E. Sluijsmans D. M. A. Driessen E. W. (2022). Putting self-regulated learning in context: integrating self-, co-, and socially shared regulation of learning. Med. Educ. 56, 29–36. doi: 10.1111/medu.14566, 33988857 PMC9291108

[ref14] Chen J. Chu L. Pan X. Chen X. (2024). Development and application of group emotional awareness tool in shared regulation: based on a large language model technical framework (in Chinese). J. Distance Educ. 42:79.

[ref15] Chen X. Hu Y. Zhang L. (2021). Group awareness in shared regulated learning: roles and mechanisms (in Chinese). J. Distance Educ. 39, 34–44. doi: 10.15881/j.cnki.cn33-1304/g4.2021.05.004

[ref16] Chen X. Luo C. Zhang J. (2019). Shared regulation: a new research and practice framework for collaborative learning (in Chinese). J. Distance Educ. 37, 62–71. doi: 10.15881/j.cnki.cn33-1304/g4.2019.01.006

[ref17] Clore G. L. Huntsinger J. R. (2007). How emotions inform judgment and regulate thought. Trends Cogn. Sci. 11, 393–399. doi: 10.1016/j.tics.2007.08.005, 17698405 PMC2483304

[ref18] Cohn C. Snyder C. Fonteles J. H. TS A. Montenegro J. Biswas G. (2025). A multimodal approach to support teacher, researcher and AI collaboration in STEM+C learning environments. Br. J. Educ. Technol. 56, 595–620. doi: 10.1111/bjet.13518

[ref19] Collins A. L. Lawrence S. A. Troth A. C. Jordan P. J. (2013). Group affective tone: a review and future research directions. J. Organ. Behav. 34, S43–S62. doi: 10.1002/job.1887

[ref20] Cortázar C. Nussbaum M. Alario-Hoyos C. Goñi J. Alvares D. (2022). The impacts of scaffolding socially shared regulation on teamwork in an online project-based course. Internet High. Educ. 55:100877. doi: 10.1016/j.iheduc.2022.100877

[ref21] Dang B. Nguyen A. Hong Y. Nguyen B.-P. T. Tran B.-N. D. (2023) Revealing the Hidden Structure of Affective States during Emotion Regulation in Synchronous Online Collaborative Learning. Honolulu, HI, USA: ScholarSpace.

[ref22] Dang B. Nguyen A. Järvelä S. (2024). Deliberative interactions for socially shared regulation in collaborative learning: an AI-driven learning analytics study. J. Learn. Anal. 11, 192–209. doi: 10.18608/jla.2024.8393

[ref23] Ekman P. Friesen W. V. (1978). Facial action coding system. Environ. Psychol. Nonverbal Behav. doi: 10.1037/t27734-000

[ref24] Elfenbein H. A. (2007). 7 emotion in organizations. Acad. Manag. Ann. 1, 315–386. doi: 10.5465/078559812

[ref25] Ez-zaouia M. Tabard A. Lavoué E. (2020). Emodash: a dashboard supporting retrospective awareness of emotions in online learning. Int. J. Hum.-Comput. Stud. 139:102411. doi: 10.1016/j.ijhcs.2020.102411

[ref26] Feidakis M. Caballé S. Daradoumis T. Jiménez D. G. Conesa J. (2014a). Providing emotion awareness and affective feedback to virtualised collaborative learning scenarios. Int. J. Contin. Eng. Educ. Life-Long Learn. 24, 141–167. doi: 10.1504/IJCEELL.2014.060154

[ref27] Feidakis M. Daradoumis T. Caballé S. Conesa J. (2014b). Embedding emotion awareness into e-learning environments. Int. J. Emerg. Technol. Learn. 9, 39–46. doi: 10.3991/ijet.v9i7.3727

[ref28] Feidakis M. Daradoumis T. Caballé S. Conesa J. Gañán D. (2013). A dual-modal system that evaluates user’s emotions in virtual learning environments and responds affectively. J. Univers. Comput. Sci. 19, 1638–1660. doi: 10.3217/jucs-019-11-1638

[ref29] Fischer F. Kollar I. Stegmann K. Wecker C. (2013). Toward a script theory of guidance in computer-supported collaborative learning. Educ. Psychol. 48, 56–66. doi: 10.1080/00461520.2012.748005, 23378679 PMC3557614

[ref30] Folkman S. Lazarus R. S. (1988). The relationship between coping and emotion: implications for theory and research. Soc. Sci. Med. 26, 309–317. doi: 10.1016/0277-9536(88)90395-4, 3279520

[ref31] Gross J. J. (2015). Emotion regulation: current status and future prospects. Psychol. Inq. 26, 1–26. doi: 10.1080/1047840X.2014.940781

[ref32] Hadwin A. F. Bakhtiar A. Miller M. (2018). Challenges in online collaboration: effects of scripting shared task perceptions. Int. J. Comput. Support. Collab. Learn. 13, 301–329. doi: 10.1007/s11412-018-9279-9

[ref33] Hadwin A. F. Järvelä S. Miller M. (2011). “Self-regulated, co-regulated, and socially shared regulation of learning,” in Handbook of Self-Regulation of Learning and Performance, eds. Barry J. Zimmerman and Dale H. Schunk vol. 30 (New York, NY: Routledge), 65–84.

[ref34] Hadwin A. Järvelä S. Miller M. (2017). “Self-regulation, co-regulation, and shared regulation in collaborative learning environments,” in Handbook of Self-Regulation of Learning and Performance, eds. Dale H. Schunk and Jeffrey A. Greene (New York, NY: Routledge).

[ref35] Harley J. M. Pekrun R. Taxer J. L. Gross J. J. (2019). Emotion regulation in achievement situations: an integrated model. Educ. Psychol. 54, 106–126. doi: 10.1080/00461520.2019.1587297

[ref36] Huang X. Zhong T. Wu H. Wang Y. Churchill E. Liu X. (2025) Linking facial recognition of emotions and socially shared regulation in medical simulation Companion Publication of the 2025 Conference on Computer-Supported Cooperative Work and Social Computing 239–243.

[ref37] Hurme T.-R. Merenluoto K. Järvelä S. (2009). Socially shared metacognition of pre-service primary teachers in a computer-supported mathematics course and their feelings of task difficulty: a case study. Educ. Res. Eval. 15, 503–524. doi: 10.1080/13803610903444659

[ref38] Isohätälä J. Järvenoja H. Järvelä S. (2017). Socially shared regulation of learning and participation in social interaction in collaborative learning. Int. J. Educ. Res. 81, 11–24. doi: 10.1016/j.ijer.2016.10.006

[ref39] Isohätälä J. Näykki P. Järvelä S. (2020). Cognitive and socio-emotional interaction in collaborative learning: exploring fluctuations in students’ participation. Scand. J. Educ. Res. 64, 831–851. doi: 10.1080/00313831.2019.1623310

[ref40] Isohätälä J. Näykki P. Järvelä S. Baker M. J. (2018). Striking a balance: socio-emotional processes during argumentation in collaborative learning interaction. Learn. Cult. Soc. Interact. 16, 1–19. doi: 10.1016/j.lcsi.2017.09.003

[ref41] Jablansky S. (2020). Relational Reasoning and Socially Shared Regulation of Learning in Collaborative Problem Solving. College Park: University of Maryland.

[ref42] Janssen J. Bodemer D. (2013). Coordinated computer-supported collaborative learning: awareness and awareness tools. Educ. Psychol. 48, 40–55. doi: 10.1080/00461520.2012.749153

[ref43] Janssen J. Erkens G. Kirschner P. A. Kanselaar G. (2012). Task-related and social regulation during online collaborative learning. Metacogn. Learn. 7, 25–43. doi: 10.1007/s11409-010-9061-5

[ref44] Järvelä S. Hadwin A. F. (2013). New frontiers: regulating learning in CSCL. Educ. Psychol. 48, 25–39. doi: 10.1080/00461520.2012.748006

[ref45] Järvelä S. Järvenoja H. Malmberg J. Isohätälä J. Sobocinski M. (2016a). How do types of interaction and phases of self-regulated learning set a stage for collaborative engagement? Learn. Instr. 43, 39–51. doi: 10.1016/j.learninstruc.2016.01.005

[ref46] Järvelä S. Kirschner P. A. Panadero E. Malmberg J. Phielix C. Jaspers J. . (2015). Enhancing socially shared regulation in collaborative learning groups: designing for CSCL regulation tools. Educ. Technol. Res. Dev. 63, 125–142. doi: 10.1007/s11423-014-9358-1

[ref47] Järvelä S. Malmberg J. Koivuniemi M. (2016b). Recognizing socially shared regulation by using the temporal sequences of online chat and logs in CSCL. Learn. Instr. 42, 1–11. doi: 10.1016/j.learninstruc.2015.10.006

[ref48] Järvelä S. Nguyen A. Hadwin A. (2023). Human and artificial intelligence collaboration for socially shared regulation in learning. Br. J. Educ. Technol. 54, 1057–1076. doi: 10.1111/bjet.13325

[ref49] Järvenoja H. Järvelä S. (2009). Emotion control in collaborative learning situations: do students regulate emotions evoked by social challenges? Br. J. Educ. Psychol. 79, 463–481. doi: 10.1348/000709909X402811, 19208290

[ref50] Järvenoja H. Malmberg J. Törmänen T. Mänty K. Haataja E. Ahola S. . (2020). A collaborative learning design for promoting and analyzing adaptive motivation and emotion regulation in the science classroom. Front. Educ. 5:111. doi: 10.3389/feduc.2020.00111

[ref51] Järvenoja H. Törmänen T. Lehtoaho E. Turunen M. Suoraniemi J. Edwards J. (2025). Investigating peer influence on collaborative group members’ motivation through the lens of socially shared regulation of learning. Br. J. Educ. Psychol. 95, 1063–1079. doi: 10.1111/bjep.12754, 39966963 PMC12590931

[ref52] Järvenoja H. Volet S. Järvelä S. (2013). Regulation of emotions in socially challenging learning situations: an instrument to measure the adaptive and social nature of the regulation process. Educ. Psychol. 33, 31–58. doi: 10.1080/01443410.2012.742334

[ref53] Kazemitabar M. Lajoie S. P. Doleck T. (2022). A process model of team emotion regulation: an expansion of Gross’ individual ER model. Learn. Cult. Soc. Interact. 33:100612. doi: 10.1016/j.lcsi.2022.100612

[ref54] Kort B. Reilly R. Picard R. W. (2001) An affective model of interplay between emotions and learning: reengineering educational pedagogy-building a learning companion Proceedings IEEE International Conference on Advanced Learning Technologies 43–46. doi: 10.1109/ICALT.2001.943850

[ref55] Kreijns K. Kirschner P. A. Jochems W. (2003). Identifying the pitfalls for social interaction in computer-supported collaborative learning environments: a review of the research. Comput. Hum. Behav. 19, 335–353. doi: 10.1016/S0747-5632(02)00057-2

[ref56] Kwon K. Liu Y.-H. Johnson L. P. (2014). Group regulation and social-emotional interactions observed in computer supported collaborative learning: comparison between good vs. poor collaborators. Comput. Educ. 78, 185–200. doi: 10.1016/j.compedu.2014.06.004

[ref57] Lantolf J. P. Swain M. (2019). On the emotion-cognition dialectic: a sociocultural response to prior. Mod. Lang. J. 103, 528–530. doi: 10.1111/modl.12574

[ref58] Lavoué E. Kazemitabar M. Doleck T. Lajoie S. P. Carrillo R. Molinari G. (2020). Towards emotion awareness tools to support emotion and appraisal regulation in academic contexts. Educ. Technol. Res. Dev. 68, 269–292. doi: 10.1007/s11423-019-09688-x

[ref59] Leony D. Muñoz-Merino P. J. Pardo A. Delgado Kloos C. (2013). Provision of awareness of learners’ emotions through visualizations in a computer interaction-based environment. Expert Syst. Appl. 40, 5093–5100. doi: 10.1016/j.eswa.2013.03.030

[ref60] Li Y. Li X. Zhang Y. Li X. (2021). The effects of a group awareness tool on knowledge construction in computer-supported collaborative learning. Br. J. Educ. Technol. 52, 1178–1196. doi: 10.1111/bjet.13066

[ref61] Li Y. Peng Y. Chen K. Su Y. (2019). Research on the functions of CSCL learning analytics tools based on group awareness (in Chinese). Mod. Educ. Technol. 1, 72–78.

[ref62] Liu D. Ding Y. (2025). How to face the problems leading to social negative emotions: a study on emotion regulation in online collaborative learning. BMC Psychol. 13:1060. doi: 10.1186/s40359-025-03432-2, 41013807 PMC12466077

[ref63] Liu M. Liu L. Liu L. (2018). Group awareness increases student engagement in online collaborative writing. Internet High. Educ. 38, 1–8. doi: 10.1016/j.iheduc.2018.04.001

[ref64] Lobczowski N. G. (2019). Building from the Inside out: The Formation and Regulation of Emotions in Collaborative Learning. Chapel Hill, NC: The University of North Carolina at Chapel Hill.

[ref65] Malmberg J. Järvelä S. Järvenoja H. (2017). Capturing temporal and sequential patterns of self-, co-, and socially shared regulation in the context of collaborative learning. Contemp. Educ. Psychol. 49, 160–174. doi: 10.1016/j.cedpsych.2017.01.009

[ref66] Malmberg J. Järvelä S. Järvenoja H. Panadero E. (2015). Promoting socially shared regulation of learning in CSCL: progress of socially shared regulation among high- and low-performing groups. Comput. Hum. Behav. 52, 562–572. doi: 10.1016/j.chb.2015.03.082

[ref67] Mänty K. Järvenoja H. Törmänen T. (2023). The sequential composition of collaborative groups’ emotion regulation in negative socio-emotional interactions. Eur. J. Psychol. Educ. 38, 203–224. doi: 10.1007/s10212-021-00589-3

[ref68] Metallinou A. Narayanan S. (2013) Annotation and processing of continuous emotional attributes: challenges and opportunities 2013 10th IEEE International Conference and Workshops on Automatic Face and Gesture Recognition (FG) 1–8. doi: 10.1016/B978-012109890-2/50031-7

[ref69] Miele D. B. (2009). Handbook of Motivation at school. New York, NY: Routledge.

[ref70] Miller M. Hadwin A. (2015). Scripting and awareness tools for regulating collaborative learning: changing the landscape of support in CSCL. Comput. Hum. Behav. 52, 573–588. doi: 10.1016/j.chb.2015.01.050

[ref71] Näykki P. Isohätälä J. Järvelä S. (2021). “You really brought all your feelings out”–scaffolding students to identify the socio-emotional and socio-cognitive challenges in collaborative learning. Learn. Cult. Soc. Interact. 30:100536.

[ref72] Näykki P. Isohätälä J. Järvelä S. Pöysä-Tarhonen J. Häkkinen P. (2017). Facilitating socio-cognitive and socio-emotional monitoring in collaborative learning with a regulation macro script – an exploratory study. Int. J. Comput.-Support. Collaborat. Learn. 12, 251–279. doi: 10.1007/s11412-017-9259-5

[ref73] Näykki P. Järvelä S. Kirschner P. A. Järvenoja H. (2014). Socio-emotional conflict in collaborative learning—a process-oriented case study in a higher education context. Int. J. Educ. Res. 68, 1–14. doi: 10.1016/j.ijer.2014.07.001

[ref74] Ngo D. Nguyen A. Dang B. Ngo H. (2024). Facial expression recognition for examining emotional regulation in synchronous online collaborative learning. Int. J. Artif. Intell. Educ. 34, 650–669. doi: 10.1007/s40593-023-00378-7

[ref75] Panadero E. Järvelä S. (2015). Socially shared regulation of learning: a review. Eur. Psychol. 20, 190–203. doi: 10.1027/1016-9040/a000226

[ref76] Pekrun R. (1992). The impact of emotions on learning and achievement: towards a theory of cognitive/motivational mediators. Appl. Psychol. 41, 359–376. doi: 10.1111/j.1464-0597.1992.tb00712.x

[ref77] Pekrun R. (2006). The control-value theory of achievement emotions: assumptions, corollaries, and implications for educational research and practice. Educ. Psychol. Rev. 18, 315–341. doi: 10.1007/s10648-006-9029-9

[ref78] Pekrun R. (2011). “Emotions as drivers of learning and cognitive development,” in New Perspectives on Affect and Learning Technologies, eds. Calvo R. A. D’Mello S. K. (New York, NY: Springer), 23–39.

[ref79] Pekrun R. Elliot A. J. Maier M. A. (2006). Achievement goals and discrete achievement emotions: a theoretical model and prospective test. J. Educ. Psychol. 98, 583–597. doi: 10.1037/0022-0663.98.3.583

[ref80] Pekrun R. Goetz T. Frenzel A. C. Barchfeld P. Perry R. P. (2011). Measuring emotions in students’ learning and performance: the achievement emotions questionnaire (AEQ). Contemp. Educ. Psychol. 36, 36–48. doi: 10.1016/j.cedpsych.2010.10.002

[ref81] Pekrun R. Goetz T. Titz W. Perry R. P. (2002). Academic emotions in students’ self-regulated learning and achievement: a program of qualitative and quantitative research. Educ. Psychol. 37, 91–105. doi: 10.1207/S15326985EP3702_4

[ref82] Pekrun R. Perry R. P. (2014). “Control-value theory of achievement emotions,” in International Handbook of Emotions in Education, eds.Reinhard Pekrun and Lisa Linnenbrink-Garcia (Routledge).

[ref83] Peng Y. Li Y. Su Y. Chen K. Jiang S. (2022). Effects of group awareness tools on students’ engagement, performance, and perceptions in online collaborative writing: intergroup information matters. Internet High. Educ. 53:100845. doi: 10.1016/j.iheduc.2022.100845

[ref84] Phielix C. Prins F. J. Kirschner P. A. Erkens G. Jaspers J. (2011). Group awareness of social and cognitive performance in a CSCL environment: effects of a peer feedback and reflection tool. Comput. Hum. Behav. 27, 1087–1102. doi: 10.1016/j.chb.2010.06.024

[ref85] Rieffe C. Oosterveld P. Miers A. C. Meerum Terwogt M. Ly V. (2008). Emotion awareness and internalising symptoms in children and adolescents: the emotion awareness questionnaire revised. Personal. Individ. Differ. 45, 756–761. doi: 10.1016/j.paid.2008.08.001

[ref86] Rimé B. (2009). Emotion elicits the social sharing of emotion: theory and empirical review. Emot. Rev. 1, 60–85. doi: 10.1177/1754073908097189

[ref87] Rimé B. Bouchat P. Paquot L. Giglio L. (2020). Intrapersonal, interpersonal, and social outcomes of the social sharing of emotion. Curr. Opin. Psychol. 31, 127–134. doi: 10.1016/j.copsyc.2019.08.024, 31590107

[ref88] Rogat T. K. Linnenbrink-Garcia L. (2011). Socially shared regulation in collaborative groups: an analysis of the interplay between quality of social regulation and group processes. Cogn. Instr. 29, 375–415. doi: 10.1080/07370008.2011.607930

[ref89] Rosenberg E. L. (1998). Levels of analysis and the organization of affect. Rev. Gen. Psychol. 2, 247–270. doi: 10.1037/1089-2680.2.3.247

[ref90] Russell J. A. (1980). A circumplex model of affect. J. Pers. Soc. Psychol. 39, 1161–1178. doi: 10.1037/h0077714

[ref91] Russell J. A. Barrett L. F. (1999). Core affect, prototypical emotional episodes, and other things called emotion: dissecting the elephant. J. Pers. Soc. Psychol. 76, 805–819. doi: 10.1037/0022-3514.76.5.805, 10353204

[ref92] Salas E. Sims D. E. Burke C. S. (2005). Is there a “big five” in teamwork? Small Group Res. 36, 555–599. doi: 10.1177/1046496405277134

[ref93] Salian B. Narvade O. Tambewagh R. Bharne S. (2021). Speech emotion recognition using time distributed CNN and LSTM. ITM Web Conf. 40:03006. doi: 10.1051/itmconf/20214003006

[ref94] Samson A. C. Huber O. Gross J. J. (2012). Emotion regulation in Asperger’s syndrome and high-functioning autism. Emotion 12, 659–665. doi: 10.1037/a0027975, 22642342

[ref95] Scherer K. R. (2005). What are emotions? And how can they be measured? Soc. Sci. Inf. 44, 695–729. doi: 10.1177/0539018405058216

[ref96] Schoor C. Bannert M. (2012). Exploring regulatory processes during a computer-supported collaborative learning task using process mining. Comput. Hum. Behav. 28, 1321–1331. doi: 10.1016/j.chb.2012.02.016

[ref97] Schraw G. Crippen K. J. Hartley K. (2006). Promoting self-regulation in science education: metacognition as part of a broader perspective on learning. Res. Sci. Educ. 36, 111–139. doi: 10.1007/s11165-005-3917-8

[ref98] Schwarz N. Clore G. L. (1983). Mood, misattribution, and judgments of well-being: informative and directive functions of affective states. J. Pers. Soc. Psychol. 45, 513–523. doi: 10.1037/0022-3514.45.3.513

[ref99] Sheng C. Liu M. Liu G. (2019). Implementation and application of an online peer assessment system based on group awareness (in Chinese). Mod. Distance Educ. Res. 31, 104–112.

[ref100] Sinatra G. M. Broughton S. H. Lombardi D. (2014). “Emotions in science education,” in International Handbook of Emotions in Education, eds. Reinhard Pekrun and Lisa Linnenbrink-Garcia (New York, NY: Routledge), 415–436.

[ref101] Sobocinski M. Järvelä S. Malmberg J. Dindar M. Isosalo A. Noponen K. (2020). How does monitoring set the stage for adaptive regulation or maladaptive behavior in collaborative learning? Metacogn. Learn. 15, 99–127. doi: 10.1007/s11409-020-09224-w

[ref102] Tinajero Vacas C. Mayo Pais E. Villar E. Martínez-López Z. (2024). Classic and modern models of self-regulated learning: integrative and componential analysis. Front. Psychol. 15:1307574. doi: 10.3389/fpsyg.2024.1307574 PMC10958659 38524299

[ref103] Törmänen T. Järvenoja H. Mänty K. (2021). All for one and one for all – how are students’ affective states and group-level emotion regulation interconnected in collaborative learning? Int. J. Educ. Res. 109:101861. doi: 10.1016/j.ijer.2021.101861

[ref104] Ucan S. Webb M. (2015). Social regulation of learning during collaborative inquiry learning in science: how does it emerge and what are its functions? Int. J. Sci. Educ. 37, 2503–2532. doi: 10.1080/09500693.2015.1083634

[ref105] van der Meer L. Costafreda S. Aleman A. David A. S. (2010). Self-reflection and the brain: a theoretical review and meta-analysis of neuroimaging studies with implications for schizophrenia. Neurosci. Biobehav. Rev. 34, 935–946. doi: 10.1016/j.neubiorev.2009.12.004, 20015455

[ref106] Van Kleef G. A. (2009). How emotions regulate social life: the emotions as social information (EASI) model. Curr. Dir. Psychol. Sci. 18, 184–188.

[ref107] Van Kleef G. A. (2016). The Interpersonal Dynamics of Emotion. Cambridge, United Kingdom: Cambridge University Press.

[ref108] Vega R. I. (2014) The Role of Student Coping in the Socially Shared Regulation of Learning in Small Groups https://repository.arizona.edu/handle/10150/323421 (Accessed May 5, 2026).

[ref109] Volet S. Summers M. Thurman J. (2009). High-level co-regulation in collaborative learning: how does it emerge and how is it sustained? Learn. Instr. 19, 128–143. doi: 10.1016/j.learninstruc.2008.03.001

[ref110] Wang M.-T. Eccles J. S. (2013). School context, achievement motivation, and academic engagement: a longitudinal study of school engagement using a multidimensional perspective. Learn. Instr. 28, 12–23. doi: 10.1016/j.learninstruc.2013.04.002

[ref111] Wang H. Zhao N. Liu S. Xu L. (2019). A study on the characteristics of emotional experiences in pre-service teachers’ online collaborative reflection (in Chinese). E-Educ. Res. 40, 113–120. doi: 10.13811/j.cnki.eer.2019.01.014

[ref112] Weiner B. (1985). An attributional theory of achievement motivation and emotion. Psychol. Rev. 92, 548–573. doi: 10.1037/0033-295X.92.4.548, 3903815

[ref113] Winnie P.H. Hadwin A. (1998). Studying as self-regulated learning. Metacogn. Educ. Theory Pract., 277–304. doi: 10.4324/9781410602350-19

[ref114] Xolocotzin Eligio U. Ainsworth S. E. Crook C. K. (2012). Emotion understanding and performance during computer-supported collaboration. Comput. Hum. Behav. 28, 2046–2054. doi: 10.1016/j.chb.2012.06.001

[ref115] Xu W. Lou Y.-F. (2024). Changes in the socially shared regulation, academic emotions, and product performance in venue-based collaborative learning. Active Learn. High. Educ. 25, 517–535. doi: 10.1177/14697874231167331

[ref116] Yuan Q. (2022). Research on classroom emotion recognition algorithm based on visual emotion classification. Comput. Intell. Neurosci. 2022, 1–10. doi: 10.1155/2022/6453499, 35978909 PMC9377850

[ref117] Zeng H. Shu X. Wang Y. Wang Y. Zhang L. Pong T.-C. . (2021). EmotionCues: emotion-oriented visual summarization of classroom videos. IEEE Trans. Vis. Comput. Graph. 27, 3168–3181. doi: 10.1109/TVCG.2019.2963659, 31902765

[ref118] Zhang S. Li H. Hui N. Guo T. Li X. Liu Q. (2023). Group awareness from a multimodal perspective: connotation, function, and implementation path (in Chinese). E-Educ. Res. 44, 20–28. doi: 10.13811/j.cnki.eer.2023.05.003

[ref119] Zheng B. Ganotice F. A. Lin C.-H. Tipoe G. L. (2023). From self-regulation to co-regulation: refining learning presence in a community of inquiry in interprofessional education. Med. Educ. Online 28:2217549. doi: 10.1080/10872981.2023.2217549, 37243670 PMC10228301

[ref120] Zimmerman B. J. (2000). “Attaining self-regulation: a social cognitive perspective,” in Handbook of Self-Regulation, eds. Monique Boekaerts, Paul R. Pintrich, and Moshe Zeidner (San Diego, CA: Elsevier), 13–39.

